# Clinical presentation, magnetic resonance imaging characteristics, and short-term outcome of deep surgical site infection following thoracolumbar decompressive spinal surgery for intervertebral disc herniation in dogs

**DOI:** 10.3389/fvets.2025.1645491

**Published:** 2025-08-13

**Authors:** Alexandra Y K To, Giunio Bruto Cherubini, Abby Caine

**Affiliations:** ^1^Dick White Referrals, Six Mile Bottom, United Kingdom; ^2^Veterinary Medical Centre, City University of Hong Kong, Hong Kong, China; ^3^Department of Veterinary Sciences, University of Pisa, Pisa, Italy; ^4^Department of Veterinary Medicine, University of Cambridge, Cambridge, United Kingdom

**Keywords:** IVDD, thoracolumbar, surgical site infection (SSI), MRI, complication

## Abstract

**Objective:**

To characterize the clinical presentation, magnetic resonance imaging (MRI) features, and short-term outcomes of deep surgical site infection (SSI) following thoracolumbar (TL) decompressive spinal surgery for intervertebral disc herniation (IVDH) in dogs.

**Method:**

Retrospective, single-center observational study of dogs that underwent postoperative MRI and were diagnosed with culture-confirmed deep SSI after TL decompressive spinal surgery between 2017 and 2021. Medical records and MRI studies (pre- and postoperative) were reviewed.

**Results:**

Nineteen dogs were diagnosed with deep SSI among 1723 thoracolumbar decompressive surgeries (incidence: 1.1%). The median time to SSI diagnosis was 7 days (range, 2–38 days). Clinical signs included spinal hyperesthesia (100%) and neurological deterioration (36.8%). MRI revealed bilateral epaxial muscle hyperintensity (66.7%), fascial plane tracking (100%), and multifocal signal voids (89.5%) as possible differentiating features. Staphylococcus spp. were the most common isolates (52.6%). All dogs survived to discharge, with 73.7% being ambulatory; short-term follow-up, available in 14/19 cases, showed resolution of clinical signs.

**Conclusion and clinical significance:**

Deep SSI after TL spinal decompression typically presents within 2 weeks with spinal hyperesthesia. Several MRI patterns may be associated with SSI. Despite rare complications, the majority of cases had favorable short-term outcomes.

## 1 Introduction

Intervertebral disc disease (IVDD) is the leading cause of canine myelopathy, with an estimated prevalence of 0.3% for disc degeneration-related conditions (1). Among these, intervertebral disc herniation (IVDH) represents the most frequently diagnosed pathology (1, 2). Treatment decisions for IVDH depend on multiple factors. While surgical decompression (e.g., hemilaminectomy or dorsal laminectomy) remains a common approach, recent literature has re-evaluated the success rate of conservative management in select cases (3). Reported success rates of surgical management in TL IVDH range from 80–95% in dogs with intact nociception, dropping to approximately 50% in dogs with loss of nociception in the pelvic limbs and tail (4–9).

Surgical site infection (SSI) is defined as an infection acquired through a surgical incision and represents one of the most prevalent nosocomial infections in humans (10). In human patients undergoing laminectomies, the reported incidence of deep SSI is approximately 0.8% (10). Deep SSI involves infection of subfascial tissues, including muscles, intervertebral discs, epidural space, vertebral bodies, and adjacent soft tissues (11). While large-scale veterinary data on post-laminectomy SSI are limited, a single-center study documented an overall SSI rate of 0.6% following hemilaminectomy and laminectomy in dogs (12). Reported SSI rates for clean surgical procedures in veterinary medicine vary widely across institutions, ranging from 0.7 to 15.6% (13–19). These infections significantly increase patient morbidity and associated treatment costs. In dogs undergoing decompressive spinal surgery, failure to recover as expected, particularly those exhibiting initial postoperative improvement followed by acute worsening of pain or neurological deficits, warrants further investigations. While recurrent disc herniation is the most frequent cause of early clinical deterioration (20, 21), SSI remains a critical differential diagnosis. Advanced imaging (e.g., MRI or CT) is essential for SSI evaluation; however, definitive diagnostic imaging features distinguishing SSI from other postoperative complications have yet to be established in veterinary medicine.

This study aimed to characterize the clinical presentation, magnetic resonance imaging (MRI) features, and short-term outcome in dogs diagnosed with deep surgical site infection following thoracolumbar decompressive spinal surgery.

## 2 Materials and methods

This was a single-center, observational, retrospective, descriptive study. The electronic database RxWorks and departmental case logs (electronic spreadsheet) at a single referral center between January 2017 and December 2021 were reviewed. All client-owned dogs undergoing thoracolumbar decompressive surgery during this period were eligible for inclusion. Written informed consent for the use of data was obtained from all owners at the time of hospital admission, as part of standard institutional protocols.

Dogs were included in this study if (1) a diagnosis of thoracolumbar IVDH was confirmed based on MRI and surgical confirmation; (2) postoperative MRI study was performed due to unsatisfactory recovery (persistent or recurrence of pain and/or neurological deterioration); (3) all MRI studies included T2-weighted sagittal and transverse sequences of the surgical site; (4) revision surgery was performed and surgical site infection was confirmed via positive bacterial culture from an intra-operative sampling of the surgical site (swab, fluid, or tissue culture).

Dogs were excluded from the study if a surgical site infection was suspected but the bacterial culture from the deep surgical site was negative, or if MRI studies did not allow for an adequate evaluation of the site of infection. Decisions on the exclusion of MRI studies were based on a consensus between a board-certified veterinary radiologist (A. C.) and a veterinary neurology resident (A. T.).

Medical records were reviewed by a veterinary neurology resident (A. T.). The following data were recorded: signalment, neurological signs at first presentation according to the modified Frankel scale (Supplementary Table 1) (8, 22), surgical site, number of days between first MRI (M1) and second MRI (M2), clinical signs, body temperature, neurological grade at onset of SSI, results of bacterial culture, and patient neurological grade at the time of discharge and where repeated neurological examination was performed by a board-certified neurologist (or resident under direct supervision of a board-certified neurologist) at the time of re-examination at the referral hospital 4 to 6 weeks after discharge.

All MRIs were acquired using a 0.4 tesla MRI scanner (Aperto Lucent, Hitachi Medical Corporation, Tokyo, Japan). All patients were scanned under general anesthesia; different anesthetic protocols were used for premedication and induction depending on the assessment and discretion of the attending anesthetist. All patients were maintained under anesthesia with isoflurane (Isothesia 100 mg/g, Henry Schein, United States) and oxygen. The dogs were scanned in dorsal recumbency.

M1 included T2-weighted (TR range 2,500–6,077 ms; TE range 100–120 ms) images in sagittal and transverse were obtained for all dogs. Additionally, short-tau inversion recovery (STIR) sagittal images (2,550 ms; 60 ms) were reviewed when available. The slice thickness was 3.0–4.0 mm.

M2 included T2W (2500–6,077 ms; 100–120 ms) images in the sagittal and transverse were obtained for all dogs. STIR, T1-weighted (639 ms; 13 ms) (T1W), and T1W after intravenous gadolinium (0.1 mmol/kg gadobutrol, Gadovist; Bayer) administration images were obtained at the discretion of the attending diagnostic imaging specialist and neurologist. The slice thickness was 3–3.5 mm.

**Figure 1:**
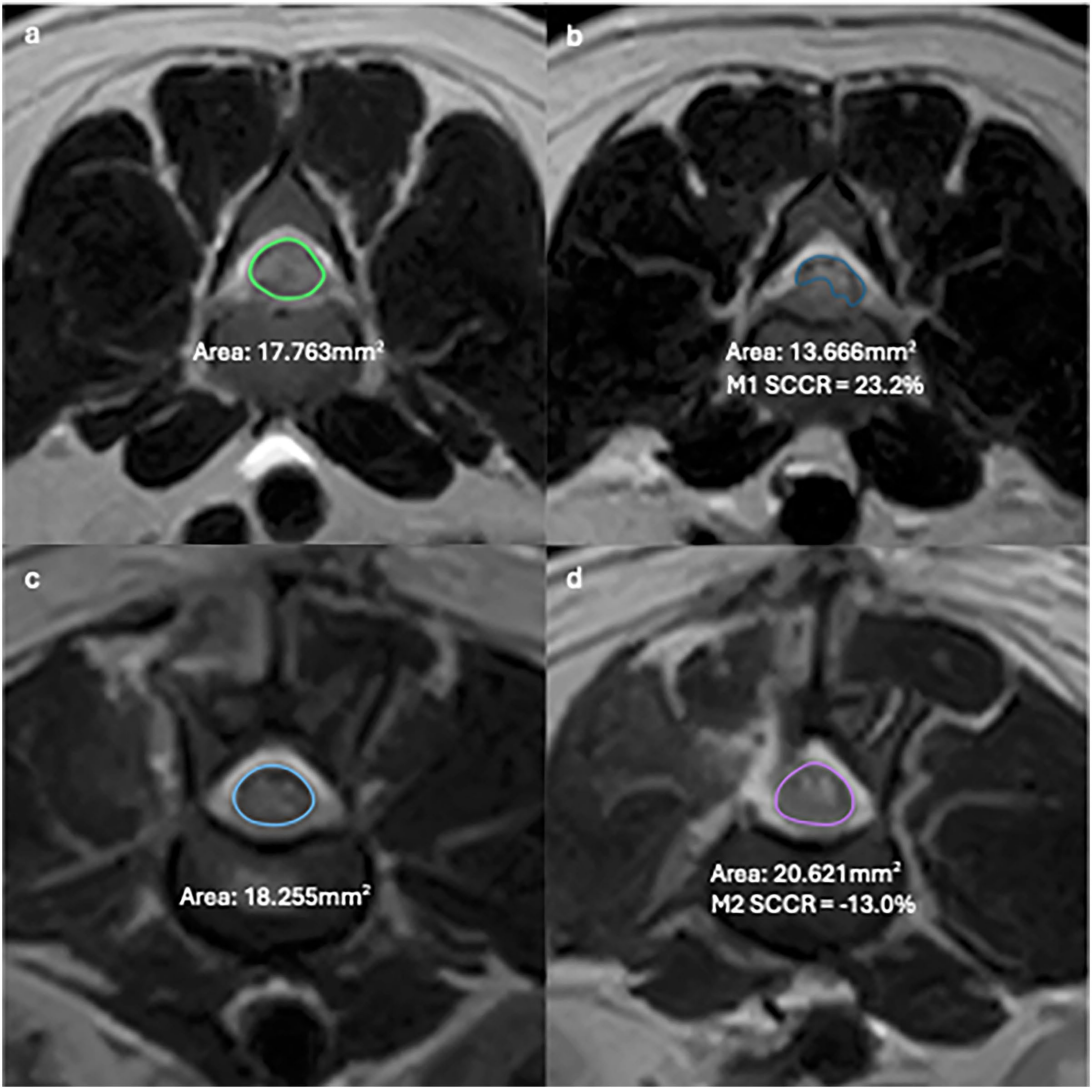
T2W Transverse MR images of the lumbar spine in a dog showing examples of SCCR in M1 between uncompressed **(a)** and compressed cord **(b)** and M2 between uncompressed **(c)** and compressed cord **(d)**.

MRI images were reviewed collaboratively by an ECVDI-certified veterinary radiologist (A. C.) and a veterinary neurology resident (A. T.) using a medical imaging viewer (OsiriX, version 13.0.2). The reviewers were blinded to the patient data and imaging reports, but not to the final diagnosis, due to the study’s inclusion criteria. The assessed MRI features are summarized in Supplementary Table 2. The change in length of spinal cord hyperintensity (T2L) and epidural attenuation (EDA) was determined by comparing measurements on the T2W sagittal sequence. The difference between M1 and M2 images was calculated and expressed as spinal cord ratio (T2LR) and epidural attenuation ratio (EDAR) using the formula [(Length in M1—Length in M2/ Length in M1)] * 100 for the respective measurement. The degree of spinal cord compression was determined by calculating the percentage of cross-sectional area (CSA) of the spinal cord at the level of maximal compression on a T2W transverse sequence and comparing it to the CSA of a distant, unaffected site. The spinal cord compression ratio (SCCR) was then calculated for both M1 and M2 using the formula [(Area of non-compressed cord—Area of maximally compressed cord)/ Area of non-compressed cord] * 100. (Figure 1).

Statistical analyses were carried out using commercially available software (IBM SPSS Statistics Desktop, Version 29.0, IBM Corporation, Armonk, New York). Categorical variables were summarized as frequency (percentage). Continuous data were tested for normality using the Shapiro–Wilk test. Normally distributed data were represented as mean, range, and standard deviation (SD); nonnormally distributed data were represented as median, range, and interquartile range (IQR).

## 3 Results

A total of 1723 cases of thoracolumbar hemilaminectomies secondary to IVDH were identified during the study period. Of those, 113 cases (6.6%) required repeated MRI and surgery within 90 days of the previous surgery. Of the 113 cases, 74 cases were excluded due to an alternative diagnosis or a negative bacterial culture. Alternative diagnoses included re-extrusion (46/1723; 2.7%), post-operative hemorrhage or hematoma formation (22/1723, 1.3%), spinal cord compression by hemostatic agent (2/1723; 0.1%), persistent intervertebral disc material (2/1723; 0.1%), and laminectomy membrane formation (1/1723; 0.1%). Of the remaining 39 cases, 20 cases (20/1723; 1.2%) were diagnosed with seroma formation with a negative bacterial culture. In total, 19 cases (19/1723; 1.1%) were identified to have positive bacterial cultures from the deep surgical site and were included in the final analysis.

### 3.1 Patient signalment

Of the 19 dogs, the majority were male (15/19, 78.9%) and neutered (12/19, 63.2%). Fourteen breeds were included, including Cocker Spaniel (2/19, 10.5%), Miniature Dachshund (2/19, 10.5%), crossbreed (3/19, 15.8%) and one of each of the following: Beagle, Border Terrier, Cavalier King Charles Spaniel, Dandie Dinmont Terrier, French Bulldog, Jack Russel Terrier, Labrador Retriever, Lhasa Apso, Pug, Spanish Water dog, Toy Poodle and Yorkshire Terrier (1/19; 5.3%). The average body weight at presentation was 12.9 kg (range 3.3 to 34.6 kg). The median age at presentation was 6 years (range 2.5 to 9 years).

### 3.2 Clinical presentation

The median neurological grade before the first surgery was 4 (range 2–4, IQR 1). The most common decompression site was T12-T13 (6/19, 31.6%), followed by T11-T12 (4/19, 21.1%), L1-L2 (2/19, 10.5%) and L4-L5 (2/19, 10.5%). One dog underwent decompression bilaterally at T11-T12 on the right and T12-T13 on the left; this dog was omitted from the analysis of lateralization of soft tissue changes. Intervertebral disc fenestration was performed on all dogs, with the majority (31.6%, 6/19) receiving fenestration at three intervertebral discs, including the herniated disc space. The hemilaminectomy site was routinely covered with a fibrillar collagen-based hemostatic agent (Lyostypt®, B. Braun) before closure at this institution. At the first surgery, all dogs received 20 mg/kg of cefuroxime intravenously 30 min before the procedure and an additional dose every 90 min during surgery, as per institutional protocol. At the second surgery, cefuroxime was withheld until after intraoperative culture samples were collected. Clinical signs associated with SSI included spinal hyperesthesia (19/19, 100%), neurological deterioration (7/19, 36.8%), superficial seroma formation (2/19, 10.5%), and urinary tract infection (2/19, 10.5%). The mean body temperature recorded at the time of SSI was 37.9°C (range 36.5–39.1°C, SD 0.74). The median number of days between the first surgery and diagnosis of SSI was 7 days (range 2–38 days, IQR 8). One dog received two 7-day courses of empirical antimicrobial therapy, 1 week apart, for the treatment of a superficial surgical site infection 2 weeks after surgery. This dog presented to the referral hospital at 38 days after surgery due to persistent spinal hyperesthesia, neurological deterioration, and superficial seroma formation. The median neurological grade at the time of SSI presentation was grade four (range 2–4, IQR 1). The neurological grade at the time of SSI presentation remained static in 12 cases (68.4%), improved by one grade in one case (5.3%), and deteriorated by one grade in four cases (21%).

### 3.3 MRI finding

MRI findings are summarized in Supplementary Tables 3, 4.

#### 3.3.1 Soft tissue

**Figure 2:**
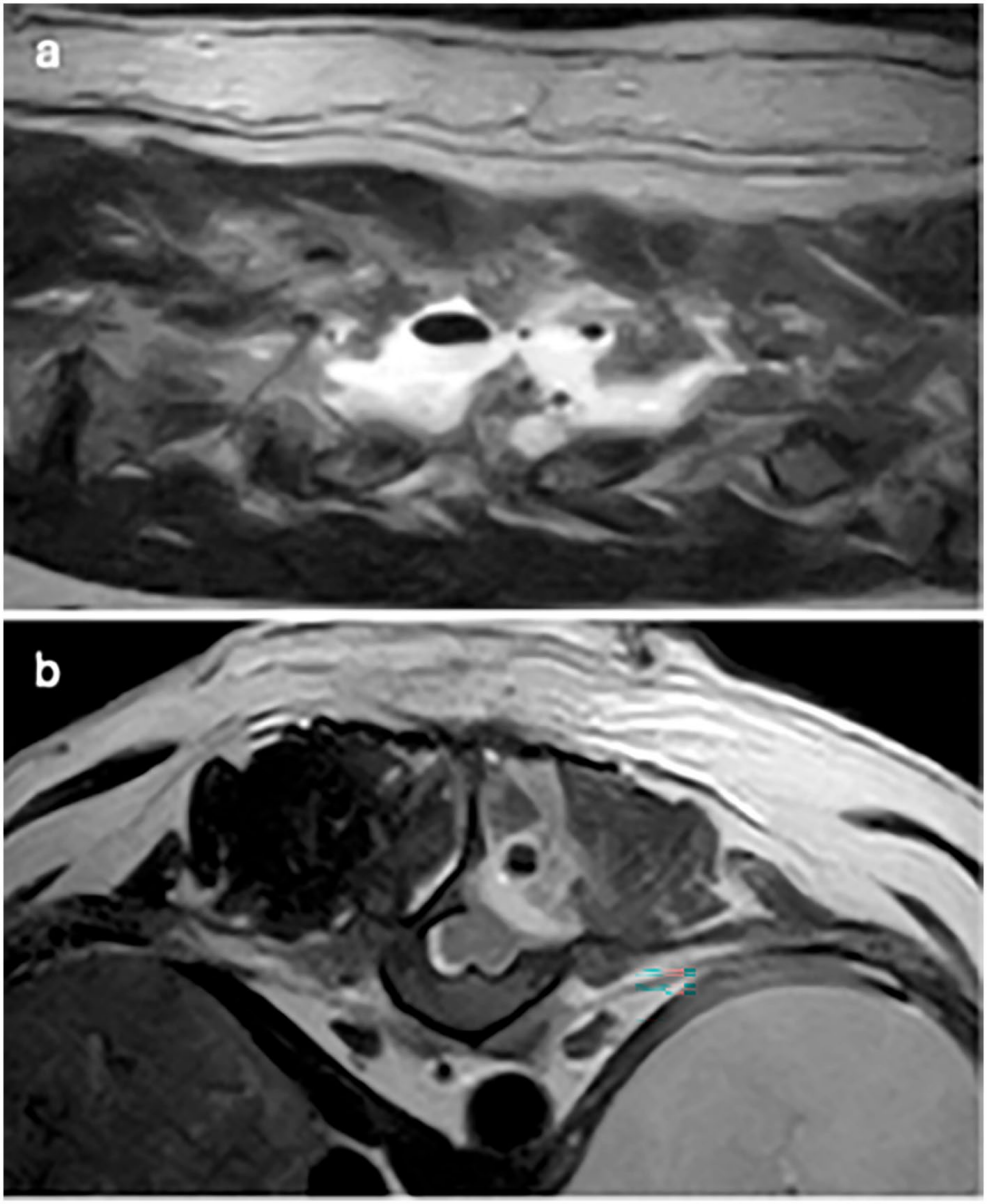
T2W images: **(a)** parasagittal image: multifocal signal voids of variable sizes within hyperintense fluid signal and **(b)** transverse image: single signal void within the epaxial muscle dorsal to the hemilaminectomy site.

**Figure 3:**
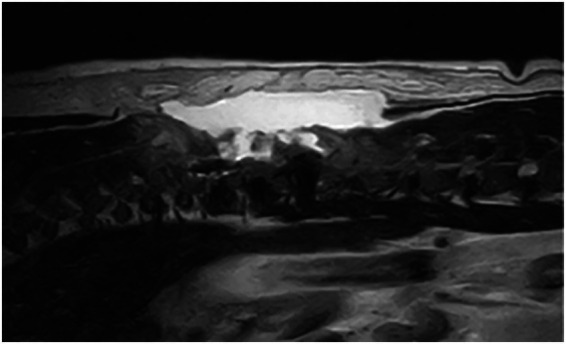
T2W sagittal after T11-T12 hemilaminectomy: large volume of accumulated hyperintense fluid.

**Figure 4:**
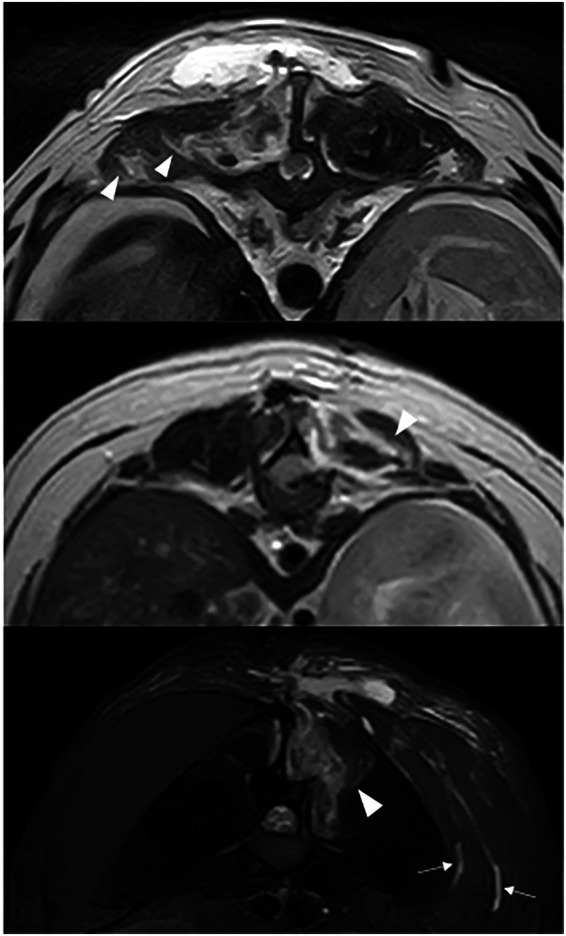
T2W **(a,b)** and STIR **(c)** transverse images: hyperintensity dissecting through the multifidus muscle (white arrow head) and subcutaneous tissue (white arrow).

**Figure 5:**
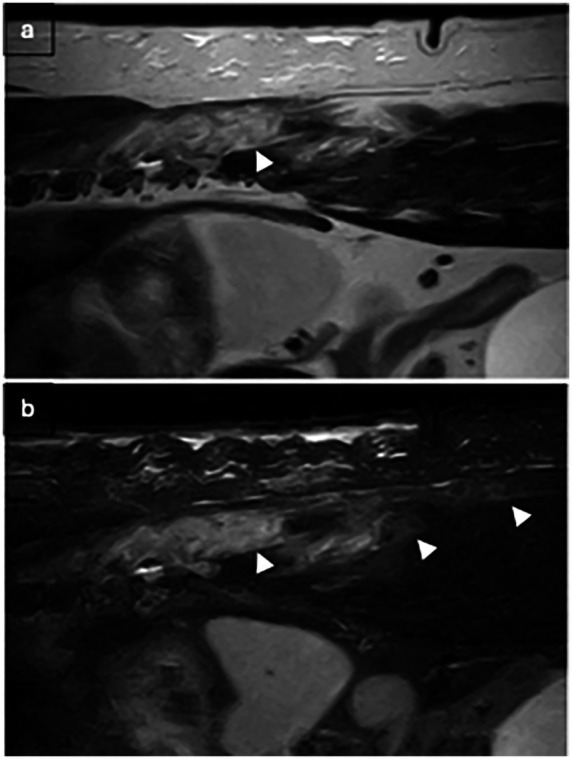
T2W **(a)** and STIR **(b)** sagittal after T9-T10 hemilaminectomy: hyperintensity dissecting through the multifidus muscle (white arrows).

Changes to the paravertebral soft tissue were appreciated in all cases postoperatively. All cases exhibited diffuse to patchy heterogeneous T2W (19 cases) and STIR (13 cases) hyperintensity in the subcutaneous soft tissue, with changes limited to the side of surgery in 3 cases (16.7%). Diffuse to patchy T2W hyperintensity of epaxial muscles was appreciated unilaterally in 6 cases (33.3%) and extended to the contralateral side in 12 cases (66.7%). Other common findings include multifocal, variably sized, rounded signal voids within the subcutaneous and muscle layers (17/19, 89.5%; Figure 2), focal accumulation of markedly T2W hyperintense material (16/19, 84%; Figure 3) and tracking of T2W and STIR hyperintensity in the epaxial muscle fascial planes (19/19, 100%; Figures 4, 5). In the case where T1W contrast images were available, rim enhancement of the ipsilateral epaxial muscle was appreciated.

#### 3.3.2 Spinal cord

The median T2LR was −3.4% (range −32.4–156.8%, IQR 46). T2LR was increased in 42.1% (8/19, mean 54.4%, range 0.5–156.8, SD 49.2) and decreased in 57.9% (11/19, mean −12.7, range −32.4–−1.2, SD 10.8). In M1, the SCCR varied between 10.2–73.5% with a mean of 29.1%, SD 16.6. In M2, SCCR varied between −36.3–39.9% with a mean of 3.32%, SD 19.1. When comparing the SCCR of M2 from M1, it was increased in 15.8% (3/19, mean 0.7, range 4.2–11.7, SD 4.0) and decreased in 84.2% (16/19, mean 8.7, range 3.2–82.8, SD 7.6). Of the 16 cases with decreased M2 SCCR, 53.3% (8/15) had a negative value, indicating an increase in spinal cord CSA. When examining epidural attenuation, the median EDAR was 5.4% (range −53.8–424.4, IQR 120). EDAR was increased in 57.9% (11/19, median 35.6%, range 0.4–424.4, IQR 94), decreased in 42.1% (8/19, median −30.1, range −53.8–1.6, IQR 49). T2W hyperintensity to normal CSF signal surrounding the spinal cord was observed in 53.6% (10/19) of cases. T2W hypointense extradural material was observed in 47.3% (9/19) of cases in the second MRI study, of those, 77.7% (7/9) were considered to be residual IVD material without causing compression, and 22.2% (2/9) were considered new material based on its shape and location. The compression from the IVD material was considered significant based on imaging in one case.

#### 3.3.3 Intervertebral disc and vertebrae

T2W and STIR hyperintensity were observed in the area of the nucleus pulposus in 52.6% (10/19) and 38.4% (5/13), respectively. Vertebral endplate T2W and STIR hyperintensity were present in 10.5% (2/19) and 15.4% (2/13) cases, respectively. In one case, these changes were associated with kyphosis at the hemilaminectomy site, widening of the remaining articular facet, and mild vertebral subluxation.

### 3.4 Clinicopathological analysis

All samples obtained from surgery underwent culture for aerobic, anaerobic, and fungal cultures. All samples yielded bacterial growth, including *Staphylococcus epidermidis* (5/19), *Staphylococcus pseudintermedius* (3/19), *Staphylococcus capitis* (1/19), *Staphylococcus haemolyticus* (1/19), *Pseudomonas aeruginosa* (2/19), *Enterococcus faecalis* (3/19), *Escherichia coli* (1/19), *Mycoplasma* species (1/19), and growth of mixed anaerobes (2/19).

### 3.5 Treatment and clinical outcome

Antimicrobials were administered to all 19 dogs after the second surgery, including cephalosporins (7/19), amoxicillin–clavulanic acid (6/19), fluoroquinolones (9/19), trimethoprim sulphonamide (6/19), metronidazole (2/19), clindamycin (3/19), tetracyclines (2/19), and/or nitrofurantoin (1/19), while awaiting culture results. After the culture results from the surgical swabs, antimicrobials were adjusted according to sensitivity. The median duration of treatment was 21 days (range: 14–84 days).

All dogs survived to discharge. At the time of discharge, 14/19 dogs (73.7%) were ambulatory, 13/19 cases (68.4%) remained neurologically stable with the resolution of spinal hyperesthesia, and 4/19 cases (21.1%) had improved by one grade. 2/19 cases (10.5%) deteriorated by one grade after repeated MRI and surgery, one was diagnosed with worsening vertebral subluxation 6 days after the first revision surgery secondary to discospondylitis, necessitating vertebral stabilization with pins and gentamicin impregnated polymethylmethacrylate (Refobacin^®^ Bone Cement R, Zimmer Biomet, United States), the other improved after adjustment of antimicrobial according to culture and sensitivity.

Short-term outcomes were available for 14 cases. At the time of follow-up, all cases were ambulatory and demonstrated resolution of spinal hyperesthesia. One case diagnosed with concurrent discospondylitis relapsed in spinal hyperesthesia after having received 3 weeks of antimicrobial therapy and 1 week after discontinuation of antimicrobial treatment due to gastrointestinal adverse reaction, clinical signs improved after re-introduction of appropriate antimicrobial for a total of 12 weeks before discontinuation.

## 4 Discussion

The incidence of deep SSI of TL decompressive surgery identified in our study was 1.1%. This aligns with previously reported rates of 0.0–1.2% (12, 18, 21) for deep SSI in canine spinal surgery, and falls within a broader 0.7–15.8% range documented for clean surgical procedures in veterinary medicine (12–19, 21). Although assessing the risk of SSI was not a primary aim of this study, prior publications from human literature have suggested an increased risk of SSI when hemostatic agents are left within the surgical site (23, 24). However, there is no consistent proof of increased risk for SSI to date (25, 26). In this study, the effect of the routine use of a fibrillar collagen-based hemostatic agent (Lyostypt^®^, B. Braun) over the hemilaminectomy site on the development of SSI is unclear, but did not result in a higher infection rate compared to the range reported earlier (1.1% versus 0.0–1.2%). Prospective, standardized studies to evaluate the effect of leaving hemostatic agents at the surgical site on the development of SSI are needed, as many factors influence SSI occurrence.

### 4.1 Clinical presentation

In our study, male dogs were over-represented (78.9%). There was no apparent age or breed predisposition. The most common clinical signs of SSI were spinal hyperesthesia (100%) and neurological deterioration (36.8%); these findings align with reported manifestations of spinal infectious conditions, including epidural empyema and paravertebral abscesses (27–31). The absence of pyrexia in this cohort was unsurprising, as non-steroidal anti-inflammatory drugs were routinely administered for postoperative analgesia in this institution. Additionally, pyrexia was an uncommon feature in naturally occurring epidural empyema or paravertebral abscesses, likely due to the localized nature of the inflammatory process (28, 29, 31). Concurrent superficial surgical complications (seroma or abscess) were uncommon, occurring in only 10.5% (2/19) of cases. In our study, 84.2% (16/19) of deep SSI cases manifested clinical signs within the early postoperative period (defined as ≤ 14 days after surgery), with a mean time to presentation of 9.5 days. This aligns with previous reports of SSI onset between 8 and 14 days (32, 33). Cases complicated by discospondylitis showed significantly delayed presentation (21 and 38 days postoperatively), suggesting a different clinical progression pattern.

### 4.2 MRI findings

T2W and STIR hyperintensity in the paravertebral soft tissue adjacent to the decompressive site, including the subcutaneous and epaxial muscles, was present in all cases. These alterations can be associated with normal post-operative oedema or associated with SSI. Changes to the epaxial muscles were common; epaxial muscle changes extended to the contralateral side in 83.3% (15/18) of cases; none of the cases had extension of soft tissue changes ventrally beyond the epaxial muscles. A prior study reported that unilateral alteration in epaxial muscles was frequently observed on MRI at surgical sites after thoracolumbar decompression surgery in the absence of SSI (34), however, the presence of T2W and STIR hyperintensity extending to the contralateral side of the surgical site was not a reported feature. This feature may therefore indicate the propagation and progression of inflammation or oedema resulting from the surgical site infection; this pattern also suggests extension via the fascial planes. The presence of small multifocal signal voids in the subcutaneous and epaxial muscle layers was present in 17 patients (89.5%). These were most likely attributed to postoperative emphysema, a common finding in the early postoperative period. However, suture artifacts, microhemorrhages, or gas production from an infectious agent could not be ruled out. Of the two cases without signal voids, one was diagnosed with discospondylitis 38 days after initial surgery, and the other 5 days after initial surgery. Interestingly, this imaging feature was only observed in a small subset of patients on follow-up MRIs in a previous study and was confined to the subcutaneous layer (34). The high frequency of signal voids observed in our population may be influenced by surgical technique as well as the temporal evolution of postoperative change; however, their increased prevalence compared to prior studies suggests they could reflect factors beyond typical postoperative artifacts. Accumulation of T2W and STIR hyperintense fluid adjacent to the surgical site was observed in 84.2% (16/19) of cases, potentially representing phlegmon, abscess, seroma, or hemorrhage. This feature has been previously described as a common finding within the first 2 weeks after surgery, representing normal postoperative changes and therefore may be considered a normal finding. The routine use of a fibrillar collagen-based haemostatic agent (Lyostypt^®^, B. Braun) over the hemilaminectomy site at this institution was also considered, as its presence could potentially influence MRI interpretation, leading to an overestimation of fluid accumulation. Previous studies have shown that collagen-based haemostatic agents typically appear as heterogeneously T2W hypo- to isointense or become hypointense after saturation with blood (35, 36). In our study, no distinct layer of a hypointense film was observed; given the frequent presence of high-signal fluid accumulation at the surgical site, it is probable that the haemostatic agent was indistinguishable from the surrounding fluid due to similar signal intensity – or that such a layer could not be resolved due to the limitation of the MRI. A novel and frequently observed feature in our cohort was the tracking of T2W hyperintensity along epaxial muscle fascial planes (present in all cases). This distinct pattern, characterized by linear signal propagation following muscle fibers and connective tissue, is consistent with phlegmonous spread, where inflammation disseminates along tissue planes. Notably, this finding has not been previously documented as a normal postoperative change, suggesting its potential utility as an SSI-specific marker. The absence of well-defined fluid collections in some of these cases further supports its classification as phlegmon rather than abscessation. When combined with other SSI features, fascial plane tracking may enhance the detection of early infections.

Longitudinal analysis of spinal cord T2W hyperintensity revealed increased lesion length in eight cases, with only four of these cases (50.0%) demonstrating concurrent neurological deterioration. Despite this discrepancy, all patients achieved positive clinical outcomes following appropriate treatment. In cases where neurological decline was observed, the lengthening of spinal cord hyperintensity may reflect SSI-associated myelitis. However, alongside the four cases without neurological deterioration, the extended hyperintensity may have represented residual injury sequelae, including persistent oedema or myelomalacia secondary to the initial IVDH. Additionally, iatrogenic contusion during surgical decompression could not be excluded as a contributing factor in these patients. The natural evolution of post-traumatic spinal cord changes over time may also account for some of the observed signal alterations. This dissociation between imaging findings and clinical signs suggests that extended T2W hyperintensity alone should not be overinterpreted in SSI cases. While it may indicate active infection when accompanied by neurological decline, it can also represent benign postoperative changes in clinically stable patients. The positive outcomes in this cohort emphasize that spinal cord signal length should not independently dictate treatment decisions in the absence of correlative neurological deficits. While successful spinal cord decompression was achieved in most cases, the presence of a small volume of residual disc material remained a common finding. This observation aligns with previous studies demonstrating that incomplete removal of extradural material occurs frequently following decompressive surgery, and neither the degree of preoperative spinal cord compression nor the volume of residual material reliably predicts neurological outcome (21, 37–39). In one case, intervertebral disc material was suspected to contribute to significant spinal cord compression in M2. Culture results in this case identified *Mycoplasma* sp., an uncommon pathogen in SSI. While this finding cannot be dismissed, the possibility of a false-positive culture must also be considered. Given the clinical presentation, the neurological signs could alternatively be attributed solely to disc re-extrusion rather than an active *Mycoplasma* infection.

MRI evaluation revealed spinal cord expansion (negative M2 SCCR) at the herniation site in 42.1% of cases (8/19), indicative of progressive oedema secondary to persistent spinal cord injury from the original disc extrusion, intraoperative manipulation, local inflammatory responses, or developing infection. Notably, while spinal cord swelling was present in 42% of cases, only 38% of these demonstrated concurrent progressive elongation of dorsal longitudinal CSF and fat signal attenuation. This partial correlation suggests that focal inflammatory changes at the injury site frequently occur without inducing widespread longitudinal perimedullary inflammation. Clinically, this observation may help differentiate focal edema from more extensive inflammatory processes such as infectious myelitis. Abnormal epidural T2W/STIR hyperintensity was observed in 52.6% (10/19) of cases, presenting a diagnostic challenge in differentiating pathological from expected postoperative changes. This imaging finding may represent infection-related pathology such as epidural empyema, infectious epiduritis, or steatitis; alternatively, it could also reflect normal postoperative inflammation. The signal characteristics alone proved insufficient for definitive diagnosis. Nucleus pulposus hyperintensity and vertebral endplate hyperintensity were present in 52 and 11% of cases, respectively. While these findings could suggest infectious processes (discitis or discospondylitis), several alternative explanations, such as iatrogenic fluid introduction during intervertebral disc fenestration, reactive bone oedema secondary to surgical manipulation, or normal postoperative inflammatory changes, must be considered. Contrary to the previous report, which described metallic susceptibility artifacts as an uncommon limitation in postoperative spinal MRI, our study found minimal image distortion from such artifacts (34). This discrepancy may reflect differences in surgical technique, as all cases in our cohort underwent standard hemilaminectomies rather than mini-hemilaminectomies. The larger surgical window in conventional hemilaminectomies likely facilitated more thorough intraoperative lavage, thereby reducing residual metallic debris from bone drilling. The absence of significant susceptibility artifacts proved particularly valuable when evaluating subtle soft tissue changes near the surgical site.

### 4.3 Treatment and outcome

The duration of antimicrobial therapy varied according to clinician preference, with nine cases receiving treatment beyond the median 21-day course. Among these, five cases demonstrated isolated intervertebral disc hyperintensity (T2W/STIR), while two had MRI-confirmed discospondylitis. Cases with disc hyperintensity alone received a median antimicrobial duration of 42 days (range: 28–84), while cases with MRI-confirmed discospondylitis received antimicrobial treatment for 84 days. This extended therapy in cases with suspected discitis or discospondylitis aligns with current practice patterns, despite the absence of standardized protocols. Notably, a large multicenter study by Van Hoof et al. (40) reported a median treatment duration of 16 weeks for infectious discospondylitis, suggesting our cohort’s management was comparatively conservative. The shorter treatment duration in our cases may have been influenced by the use of repeat surgical intervention—including debridement and irrigation—which likely reduced the infectious burden. Therapeutic decisions in our cohort appeared to be primarily guided by MRI findings and clinician preference, underscoring the need for standardized, evidence-based protocols in the management of SSIs.

The study demonstrated favorable outcomes in the majority of cases, with 73.7% (14/19) of dogs being ambulatory at the time of discharge. Notably, pre-SSI neurological status remained strongly prognostic; 71.4% (10/14) of dogs that were ambulatory prior to the detection of infection maintained ambulation. Of the five dogs that were non-ambulatory at the time of discharge, four subsequently regained ambulation during short-term follow-up. However, precise time-to-ambulation data were unavailable for retrospective analysis. One case was lost to follow-up, precluding definitive outcome assessment. These positive results support the likelihood of neurological recovery following appropriate SSI management; non-ambulation status should not be considered a negative prognostic indicator when treating postoperative spinal infections.

### 4.4 Limitations

This study has several important limitations inherent to its retrospective, single-center design. First, variability in MRI protocols across cases, particularly the inconsistent availability of STIR, T1-weighted, and contrast-enhanced sequences. This limited detailed pathological differentiation and likely reduced sensitivity for detecting subtle abnormalities. While all studies included a standardized minimum sequence protocol (T2W sagittal and transverse), the absence of additional sequences in some cases may have underrepresented certain pathological features. Second, although assessors were blinded to prior radiological reports, their awareness of the inclusion criteria and final SSI diagnoses potentially introduced interpretation bias. Third, the descriptive nature of the analysis precludes definitive conclusions about causality or treatment efficacy. These limitations highlight the need for prospective, multi-center studies with standardized imaging protocols and fully blinded evaluations to validate our findings.

The study’s reliance on positive bacterial cultures as an inclusion criterion introduced inherent selection bias that warrants careful consideration. By design, this approach necessarily excluded culture-negative infections that might have resulted from prior antibiotic administration, fastidious organisms, or lower bacterial burdens. The selection bias introduced by culture-based inclusion criteria preferentially identified infections with higher microbial loads and more advanced pathological changes, potentially creating a spectrum bias that overrepresents severe MRI manifestations. The requirement for microbiological confirmation, while providing diagnostic certainty for the study’s primary aims, may consequently limit the generalizability of our findings to earlier or more subtle presentations of deep SSI. These observations highlight the need to develop more inclusive diagnostic criteria for future investigations, potentially incorporating histopathological confirmation to capture the full spectrum of postoperative infections.

The findings of this study must be contextualized by its notable lack of grade 1 neurological cases, which may create a potentially significant spectrum bias toward more favorable outcomes. This limitation carries important clinical implications. The development of progressive myelomalacia, seen primarily in grade 1 patients, often presents with clinical deterioration that may overlap with deep SSI manifestations. In practice, such cases frequently undergo euthanasia without repeat imaging, systematically excluding potential neurological deterioration brought about by infection from our analysis.

The small sample size and absence of a control group in this study present important interpretative limitations, as some observed MRI changes could represent normal postoperative variation rather than definitive signs of infection. This diagnostic challenge is compounded by the ethical and practical constraints of obtaining routine postoperative MRI scans in asymptomatic patients solely for research comparisons. While our findings demonstrated MRI features consistent with prior reports of spinal infections, the lack of standardized controls necessitates cautious interpretation (27–31). To partially address this limitation, we compared our results with published data on non-infected postoperative cases, revealing several potentially discriminatory features. Notably, the combination of bilateral epaxial muscle involvement, tracking of hyperintensities through fascial planes, and multifocal signal voids within the epaxial muscle layer appeared more characteristic of infection than normal postoperative change (34). Nevertheless, these observations require validation through larger, prospective studies incorporating both infected and carefully matched non-infected control populations.

The imaging evaluations in this study were conducted through consensus review by two blinded observers: a board-certified veterinary radiologist and a neurology resident. To maximize objectivity, we employed dichotomous criteria for key findings wherever feasible. While this approach reduced reliance on subjective interpretation, inherent variability may have persisted due to differences in reviewer experience and training backgrounds. The potential influence of observer expertise on image interpretation, though recognized as an important consideration, was not formally evaluated as part of this study design. This methodological limitation is partially mitigated by the use of standardized evaluation criteria and consensus-based decision making.

## 5 Conclusion

In conclusion, this study highlights the importance of vigilant postoperative monitoring during the initial two-week period when most SSIs become apparent, and that MRI serves as a valuable diagnostic tool for differentiating SSIs from other causes of postoperative deterioration. The identified MRI characteristics, including paraspinal soft tissue changes, epidural hyperintensity, and fascial plane tracking, may enhance diagnostic confidence even in culture-negative cases, potentially enabling more judicious antimicrobial use.

## Data Availability

The original contributions presented in the study are included in the article/Supplementary material, further inquiries can be directed to the corresponding author.
