# Supplementary material for: Clinical presentation, magnetic resonance imaging characteristics, and short-term outcome of deep surgical site infection following thoracolumbar decompressive spinal surgery for intervertebral disc herniation in dogs
Source: Front Vet Sci. 2025 Aug 13;12:1645491. doi: 10.3389/fvets.2025.1645491 (PMC12380707; doi:10.3389/fvets.2025.1645491)
Supplement: Supplementary file 1 [file Table_1.docx]

| Modified Frankel Score | Neurological status |
| --- | --- |
| 5 | Spinal hyperesthesia only |
| 4 | Ambulatory paraparesis |
| 3 | Non-ambulatory paraparesis |
| 2 | Paraplegia with intact nociception in the pelvic limbs and/or tail |
| 1 | Paraplegia with absent nociception in the pelvic limbs and tail |

Table 1. Modified Frankel Score^8,22^

| Structure evaluated | Features identified | Sequence and plane evaluated |
| --- | --- | --- |
| Subcutis Layer | Prescence or absence of abnormal hyperintensity | T2 Sagittal, T2 Transverse, STIR Sagittal* |
| Epaxial Muscle Layer | Prescence or absence:   - Abnormal hyperintensity - Tracking of hyperintensity through muscle planes - Signal voids - Homogeneous region of hyperintensity | T2 Sagittal, T2 Transverse, STIR Sagittal* |
| Spinal cord | - Length of spinal cord hyperintensity - Ratio of maximum spinal cord compression | T2 Sagittal  T2 Transverse |
| Epidural space | - Length of attenuation of dorsal epidural space and CSF column - Abnormal rim of epidural hyperintensity | T2 Sagittal  T2 Transverse  STIR Transverse* |
| Intervertebral Disc | - Abnormal hyperintensity of IVD | T2 Sagittal  STIR Sagittal*  T1 Post-contrast Sagittal* |
| Vertebral Endplate | - Abnormal hyperintensity of vertebral endplate | T2 Sagittal  STIR Sagittal*  T1 Post-contrast Sagittal* |

Table 2. Representing the evaluated structures, the observed MRI features and the sequences evaluated for each feature. *Sequence evaluated if available

| Case no. | Subcutaneous hyperintensity | Muscle hyperintensity | T2 Signal Voids | Fluid Pocket | Hyperintensity tracking through fascial plane | Hyperintensity around spinal cord | IVD T2 Hyperintensity (compared M1) | Vertebral endplate T2 Hyperintensity | Presence of residual disc material | Presence of new disc material |
| --- | --- | --- | --- | --- | --- | --- | --- | --- | --- | --- |
| 1 | + | + | + | + | + | - | - | - | + | - |
| 2 | + | + | + | - | + | - | - | + | + | + |
| 3 | + | + | + | + | + | - | - | - | - | - |
| 4 | + | + | + | + | + | - | + | - | - | - |
| 5 | + | + | + | + | + | + | - | - | - | - |
| 6 | + | + | + | + | + | - | - | - | - | - |
| 7 | + | + | + | + | + | + | + | - | - | - |
| 8 | + | + | + | + | + | + | - | - | - | - |
| 9 | + | + | + | - | + | - | + | - | - | + |
| 10 | + | + | + | + | + | + | - | - | + | - |
| 11 | + | + | + | + | + | + | + | - | - | - |
| 12 | + | + | + | + | + | + | - | - | - | - |
| 13 | + | + | + | + | + | - | + | - | + | - |
| 14 | + | + | + | - | + | + | + | - | + | - |
| 15 | + | + | + | + | + | + | + | - | + | - |
| 16 | + | + | + | + | + | + | + | - | + | - |
| 17 | + | + | + | + | + | - | + | - | - | - |
| 18 | + | + | - | + | + | + | + | + | - | - |
| 19 | + | + | - | + | - | - | - | - | - | + |

Table 3. Summary of the categorical MRI findings. +: positive, -: negative.

| Case no. | T2LR | M1 SCCR | M2 SCCR | M2 SCCR – M1 SCCR | EDAR |
| --- | --- | --- | --- | --- | --- |
| 1 | 86.56% | 26.72% | 0.41% | 26.31% | 104.92% |
| 2 | 55.98% | 45.39% | 39.86% | 5.54% | -45.29% |
| 3 | -8.46% | 22.90% | 0.01% | 22.90% | -2.48% |
| 4 | -20.80% | 73.55% | -9.22% | 82.77% | 6.56% |
| 5 | -9.86% | 10.18% | 16.00% | -5.82% | 0.44% |
| 6 | 156.81% | 35.89% | -15.46% | 51.35% | 220.00% |
| 7 | 36.29% | 29.98% | 26.82% | 3.16% | 277.58% |
| 8 | 50.74% | 46.53% | -10.31% | 56.84% | 37.31% |
| 9 | -25.61% | 21.96% | 26.14% | -4.18% | -53.77% |
| 10 | -23.33% | 23.62% | -3.25% | 26.87% | -53.28% |
| 11 | -4.17% | 16.71% | 28.42% | -11.71% | -1.63% |
| 12 | -3.38% | 43.51% | 15.74% | 27.76% | 82.38% |
| 13 | 0.46% | 13.07% | -15.12% | 28.19% | 200.00% |
| 14 | 36.28% | 52.33% | 15.66% | 36.67% | -48.39% |
| 15 | -5.91% | 22.44% | 0.03% | 22.41% | -6.38% |
| 16 | -4.43% | 23.19% | -12.96% | 36.16% | -14.89% |
| 17 | -1.24% | 17.09% | -36.29% | 53.38% | 5.39% |
| 18 | 11.89% | 10.60% | 2.43% | 8.17% | 424.39% |
| 19 | -32.39% | 17.19% | -5.92% | 23.10% | 33.88% |

Table 4. Summary of measured ratios. T2LR: Spinal cord hyperintensity ratio; SCCR: spinal cord compression ratio; EDAR: epidural attenuation ratio.
